# Sellick maneuver assisted real-time to achieve target force range in simulated environment—A prospective observational cross-sectional study on manikin

**DOI:** 10.1371/journal.pone.0227805

**Published:** 2020-02-11

**Authors:** Hwan Ing Hee, Chiong Ling Wong, Olivia Wijeweera, Rehena Sultana, Ban Leong Sng

**Affiliations:** 1 Department of Paediatric Anaesthesia, KK Women’s and Children’s Hospital; Duke NUS Medical School, Singapore; 2 Department of Anaesthesiology, Khoo Teck Puat Hospital, Singapore; 3 Department Biostatistics, Duke NUS Medical School, Singapore; 4 Department of Women’s Anaesthesia, KK Women’s and Children’s Hospital; Duke NUS Medical School, Singapore; AOU Policlinico Vittorio Emanuele, ITALY

## Abstract

A force sensor system was developed to give real-time visual feedback on a range of force. In a prospective observational cross-section study, twenty-two anaesthesia nurses applied cricoid pressure at a target range of 30–40 Newtons for 60 seconds in three sequential steps on manikin: Group A (step 1 blinded, no sensor), Group B (step 2 blinded sensor), Group C (step 3 sensor feedback). A weighing scale was placed below the manikin. This procedure was repeated once again at least 1 week apart. The feedback system used 3 different colours to indicate the force range achieved as below target, achieve target, above target. Significantly higher proportion of target cricoid pressure was achieved with the use of sensor feedback in Group C; 85.9% (95%CI: 82.7%-88.7%) compared to when blinded from sensor in Group B; 31.3% (95%CI: 27.4–35.4%). Cricoid force achieved blind (Group B) exceeded force achieved with feedback (Group C) by a mean of 8.0 (95%CI: 5.9–10.2, p<0.0001) and 6.2 (95%CI:4.1–8.3, p< 0.0001) Newtons in round 1 and 2 respectively. Weighing scale read lower than corresponding force sensor by a mean of 8.4 Newtons (95% CI: 7.1–9.7, p<0.0001) in group B and 5.8 Newtons (95% CI: 4.5–7.1, p<0.0001) in Group C. Force sensor visual feedback system enabled application of reproducible target cricoid pressure with less variability and has potential value in clinical use. Using weighing scale to quantify and train cricoid pressure requires a review. Understanding the force applied is the first step to make cricoid pressure a safe procedure.

## Introduction

Cricoid pressure is a common anaesthetic procedure during rapid sequence induction recommended by anaesthesia professional bodies [1,2], with more than 92% of British anaesthetists [3] using it in all emergency operations. Cricoid pressure is also amongst the first procedure skill taught to trainee anesthetists and assessed for competency in the United Kingdom and Ireland [4]. Today, cricoid pressure remains a relevant maneuver in clinical practice and deserves more studies to better understand it and to improve its performance by operators.

Currently, the force applied is based on prior experience and training. Training strategies of cricoid pressure are varied and include the use of weighing scale and laryngotracheal models [5–8], force sensor [9], pressurized syringe [10], and pain-induced digital pressure on nasal alar [7,9,11]. Previous studies have reported that knowledge about cricoid pressure, the applied cricoid pressure and technique were either inappropriate or variable in the technique [5–10, 12–13].

There are considerable safety concerns regarding poorly applied cricoid pressure which can lead to difficult mask ventilation, direct laryngoscopy or supraglottic airway device insertion. Nevertheless, the blind practice of cricoid pressure without real-time measurement is still the standard practice. Studies on the use of real-time force sensing feedback to guide the application of force in clinical situations is limited, tools studied to aid cricoid force application are bulky and not adopted in routine clinical setting [11, 14,15].

In the last decade, performance feedback technology in vital procedures, such as cardiopulmonary resuscitation (CPR) has shown that real-time sensing of objective key metrics and feedback during resuscitation resulted in better quality of CPR, increase in survival and favorable functional outcomes after cardiac arrest [16, 17, 18]. We propose the use of real time objective feedback of cricoid pressure to guide the application of recommended target range of cricoid pressure.

In this prospective exploratory study, we developed a force sensor system to measure force and give real-time visual feedback on the range of force attained during cricoid pressure. The primary aim is to evaluate the efficacy of a force sensor system that gives real time visual feedback during simulated cricoid pressure application on manikin by anaesthesia nurses. The primary endpoint was the percentage proportion of force attained within the target range. Secondarily, we also compared force measured with sensor and force transmitted inferiorly from manikin onto the weighing scale.

## Methods

### Study design

The study was a prospective observational cross-sectional study performed in a simulated environment on an airway manikin. Institutional ethics approval (Singhealth CIRB 2015/2551) was obtained. The manuscript adhered to STROBE guidelines.

### Study setting and population

Participants were anaesthesia Nurses from the Major Operating Theatre, KK Women’s and Children’s Hospital, recruited between July to October 2014 with written informed consent. Exclusion criteria were back pain and pregnancy state.

### Study protocol

#### Subjects

Anaesthesia nurses are nurses who assist anesthesiologist in airway management in the operating theatre and in recovery. The nurses routinely provide cricoid pressure to patients during rapid sequence induction. Participants received a case scenario to apply cricoid pressure using a three fingers technique on an airway manikin within a target range of 30–40 Newtons for 60 seconds in three sequential activities as described below:

Step 1: Group A (Blinded), directly on cricoid region of the manikin without a force sensor.
Step 2: Group B (Blinded sensor), directly on a force sensor placed over cricoid region of the manikin but with feedback display covered.
Step 3: Group C (Feedback sensor), directly on a force sensor placed over cricoid region of the manikin with feedback display.

All participants received standardized instruction, demonstration and hands on trial prior to data collection. All participants completed the sequential activities with a 5-minutes rest between each activity. We repeated the procedure testing a second time for each participant at least 1 week later, denoted as round 1 for the first procedure and round 2 for the second procedure. This was to evaluate the repeatability of the performance of the participants with the use of the feedback system. Group A was included in the experiment design to allow evaluation of repeatability of performance of the anaesthesia nurse when cricoid pressure is applied blind in the natural setting.

#### Manikin

Laerdal^®^ Airway Management Trainer (Laerdal^®^ Airway Management Trainer, Laerdal Medical, Stavanger, Norway) with an upper torso and head manikin was placed on a calibrated weighing scale. The whole unit was then placed on a trolley at a height similar to the operating table to reproduce real-life situation in the operating room.

#### Weighing scale

The weighing scale used was Seca 717 electronic baby scale (Seca GmbH, Germany) with graduation of 1gram.

#### The force sensor system with visual feedback

**Fig 1:**
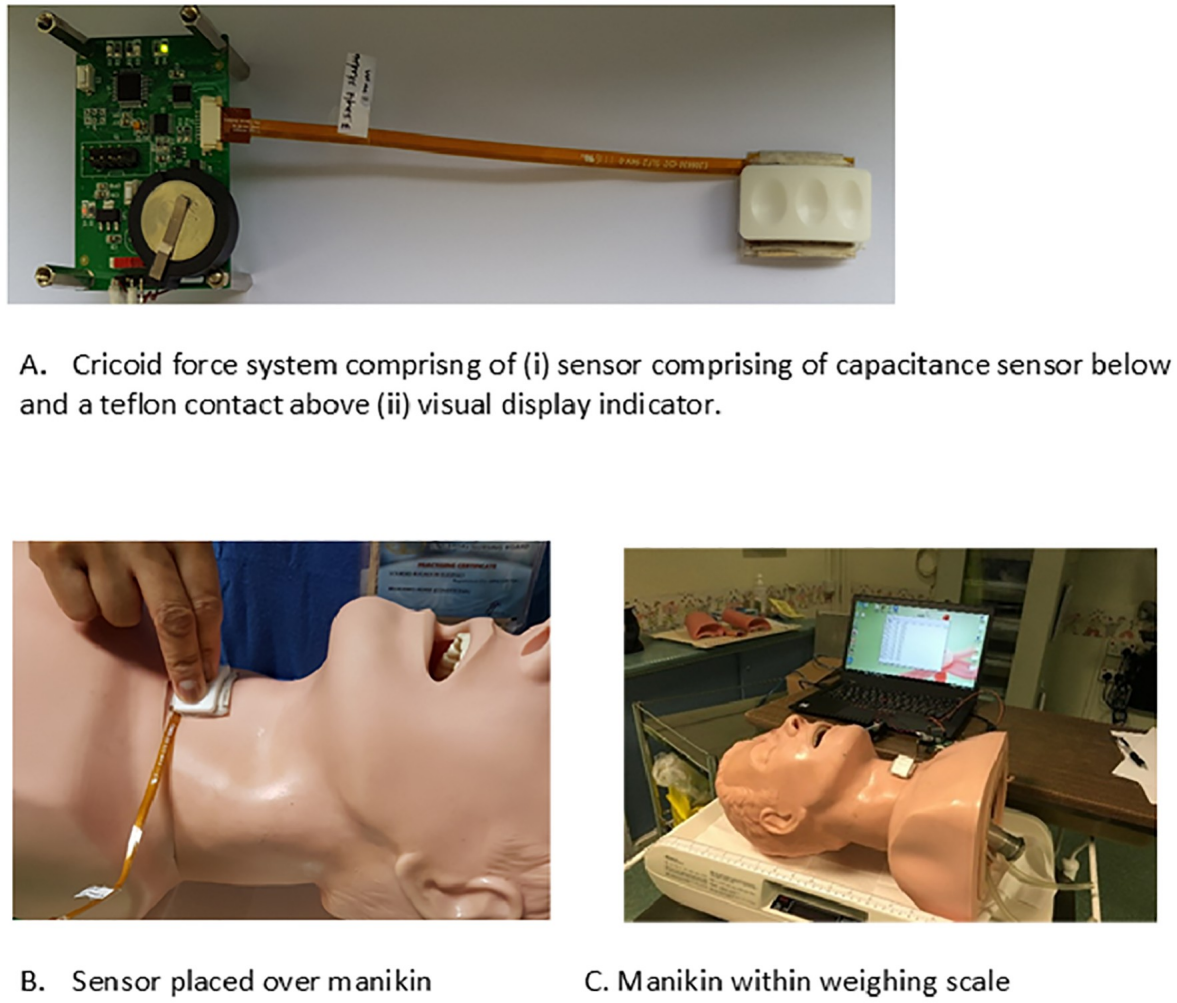
Illustration of the cricoid force sensor system (a) and experiment set up with sensor placed over manikin (b) and manikin placed above weighing scale (c).

The force sensor system consisted of an upper preformed convex interface contact layer and a lower capacitance microelectromechanical system force sensor array. This sensor is connected to a visual display and a computer. The display unit was calibrated to give three distinct colours to indicate three force range: below, at or above our target range. Here, the target range was 30 to 40 Newtons (N). The force sensor measured the applied contact force on the sensor and the display unit gave real-time visual feedback to indicate the force achieved. The force sensor system and the experimental setup is illustrated in Fig 1. The force sensor was tested and characterized in the laboratory using force gauge and motorized force tester, Chatillon LMTC DFE 2–100 (Ametek Inc, Pennsylvania, United States) as described by Ning [19] and showed an accuracy of 1.5–2.5 Newtons for a force range of 10–40 Newtons. The numerical value of cricoid force was captured by the force sensor and transferred to the computer system.

### Measurement and performance data acquisition

Force transmitted downwards to the weighing scale (WS) were recorded for all three groups A, B and C. In Group B and Group C, cricoid force applied on the surface of force sensor (SS) was recorded.

The force data were expressed as Newtons. Data at every 5 seconds were extracted for analysis, thus for each set of 60 seconds pressure application, there were 12 data points. Force measured from WS was converted into Newtons to allow comparison with SS. The change of 1-kilogram unit was taken as 10 Newtons.

### Outcome

Primary endpoint was the percentage proportion of data points of force data that was attained within the target range of 30–40 N. Secondary endpoints were repeatability of performance and comparison of force measured using SS (force applied at surface) and weighing scale WS (force transmitted inferiorly).

### Statistical analysis

Force measured based on WS and SS were continuous data. Force measured were expressed as mean (standard deviation (SD)) or mean with 95% confidence interval (95%CI). Force measured were compared using two sample Student’s t-test between group B and group C for both WS and SS. Force achieved target range was expressed as frequency (percentage) for both group B and C for SS. Difference in achieving target force was tested using Fisher’s exact test. We also compared the force applied for round 1 and round 2 using Student’s T-test for both WS and SS measurements separately. A p value < 0.05 was considered as statistically significant. All tests were two sided. Analysis was done using SAS version 9.4.

Study was adequately powered based with a total of 468 (234 X 2) readings in two rounds on the following assumptions: proportion of target force achieved by group B as 40% and by group C as 65% (conservative i.e. at least a difference between two groups in achieved target force), level of significance as 5% and power as 90% and McNemar test for paired comparison. There were at least 496 (248 X 2) readings, hence the study was adequately powered for the primary objective. Each nurse required to press for 60 seconds. Readings were taken every at every 5 seconds interval. Hence, we needed 22 nurses to achieve the calculated readings on each group.

## Results

Twenty-two female nurses were enrolled and all completed the 2 rounds of study. The mean (SD) age of nurses was 32.2 (6.7) years, with average (SD) 6.5 (4.3) and 5.8 (3.7) years of experience in anaesthesia and cricoid pressure use respectively. The participants repeated round 1 and round 2 of the procedures at a mean (SD) time interval of 64.6 (42.1) days apart.

There were 264 readings for both round 1 and round 2 in group B; and 248 and 264 readings for round 1 and 2 in Group C. The percentage of readings within target range was 70 (26.5%) and 88 (33.3%) for Group B in round 1 and 2 respectively and 201 (81.1%) and 233 (88.3%) for Group C in round 1 and 2 respectively, the difference in proportion is statistically significant. (p < 0.0001).

### Outcome between blind and feedback

Cricoid pressure performance using WS and SS was summarized in Table 1. There is no significant difference in the force reading between the first and second round in each group, illustrating repeatability in the study for all groups.

**Table 1:** Performance of cricoid pressure. Continuous data is presented as mean ± SD (95% CI). p–values are comparing difference between round 1 and round 2 within each group.

Force Applied on Device (Newtons)	Round 1 Mean±SD (95%CI)	Round 2 Mean±SD (95%CI)	Difference Mean (95% CI)	P- value
**Weighing scale (WS)**				
Group A	39.6±3.4 (32.8, 46.3)	37.7±3.4 (31, 44.5)	1.8 (-6.8, 10.4)	0.6749
Group B	33.4±3.4 (26.7, 40.2)	29.8±3.4 (23.1, 36.6)	3.6 (-5.0, 12.2)	0.4154
Group C	28.7±3.4 (21.9, 35.4)	25.4±3.4 (18.7, 32.2)	3.2 (-5.4, 11.8)	0.4634
**Sensor Scale (SS)**				
Group B	39.8±1.4 (37.0, 42.7)	39.5±1.4 (36.6, 42.3)	0.3 (-1.8, 2.5)	0.7487
Group C	31.8±1.5 (28.9, 34.7)	33.3±1.4 (30.5, 36.1)	-1.5 (-3.6, 0.7)	0.1776

The difference in force between groups in round 1 and round are summarized in Table 2.

**Table 2:** Difference in performance between groups using both weighing scale (WS) and sensor scale (SS). Continuous data is presented as mean + SD (95% CI). p–values are comparing difference between round 1 and round 2 between groups.

*Group*	*Difference (95%CI)*	*P—value*
***Weighing scale (WS)***		
*Group A–Group B*		
*Round 1*	6.2 (4.5, 7.9)	< 0.0001
*Round 2*	7.9 (6.3, 9.5)	< 0.0001
*Group A–Group C*		
*Round 1*	10.9 (9.2, 12.6)	< 0.0001
*Round 2*	12.3 (10.7, 13.9)	< 0.0001
*Group B–Group C*		
*Round 1*	4.8 (3.0, 6.5)	< 0.0001
*Round 2*	4.4 (2.8, 6.0)	< 0.0001
***Sensor Scale (SS)***		
*Group B–Group C*		
*Round 1*	8.0 (5.9, 10.2)	< 0.0001
*Round 2*	6.2 (4.1, 8.3)	< 0.0001

**Fig 2:**
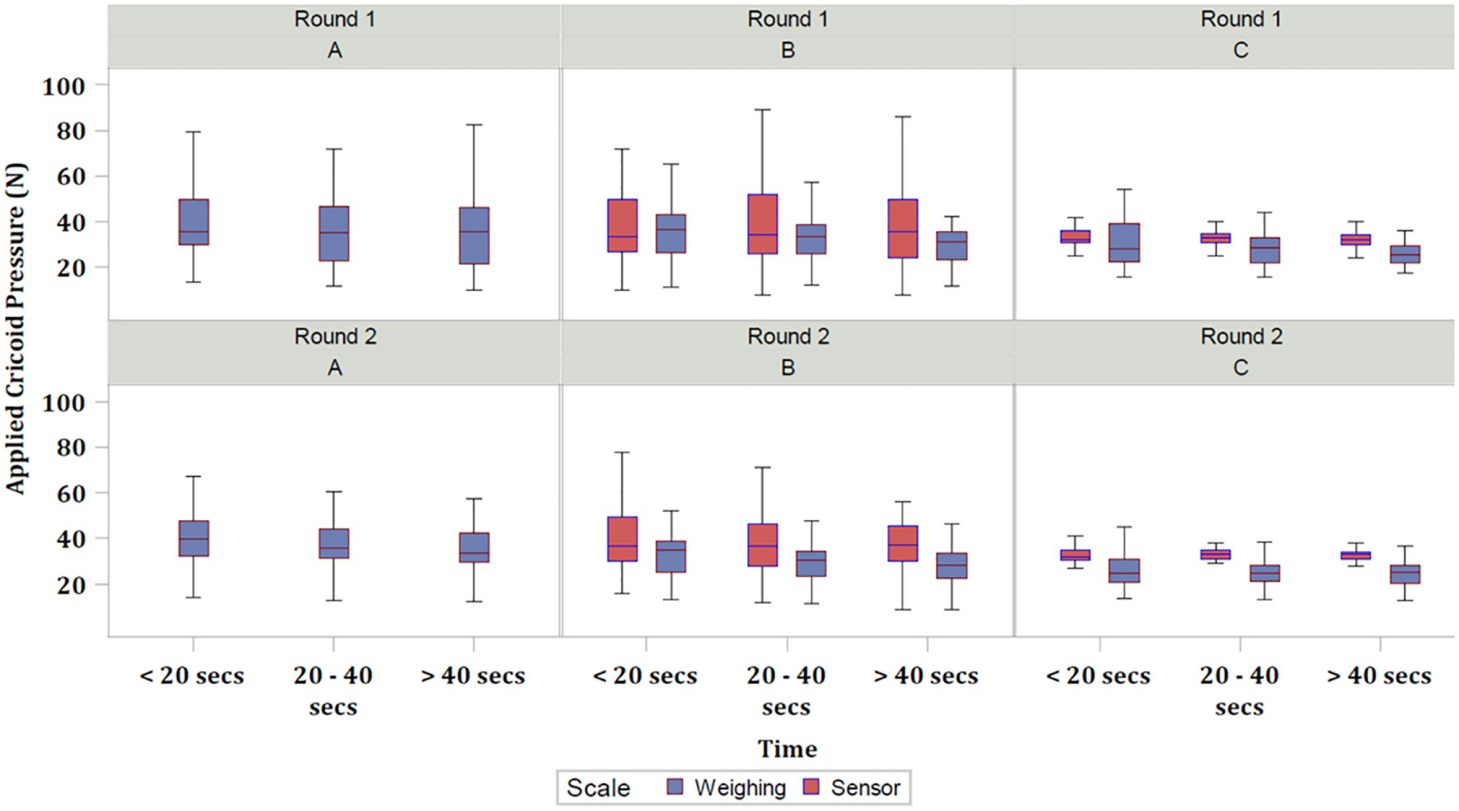
Distribution of cricoid pressure over time based on rounds and groups. Cricoid pressure measured using weighing scale (WS) and force sensor scale (SS).

The performance of cricoid pressure over time in each activity is illustrated in Fig 2. Each 60 seconds activity was divided into three 20 seconds intervals. With the use of sensor in Group C, the cricoid force achieved is found to be consistent over time within each activity.

**Fig 3:**
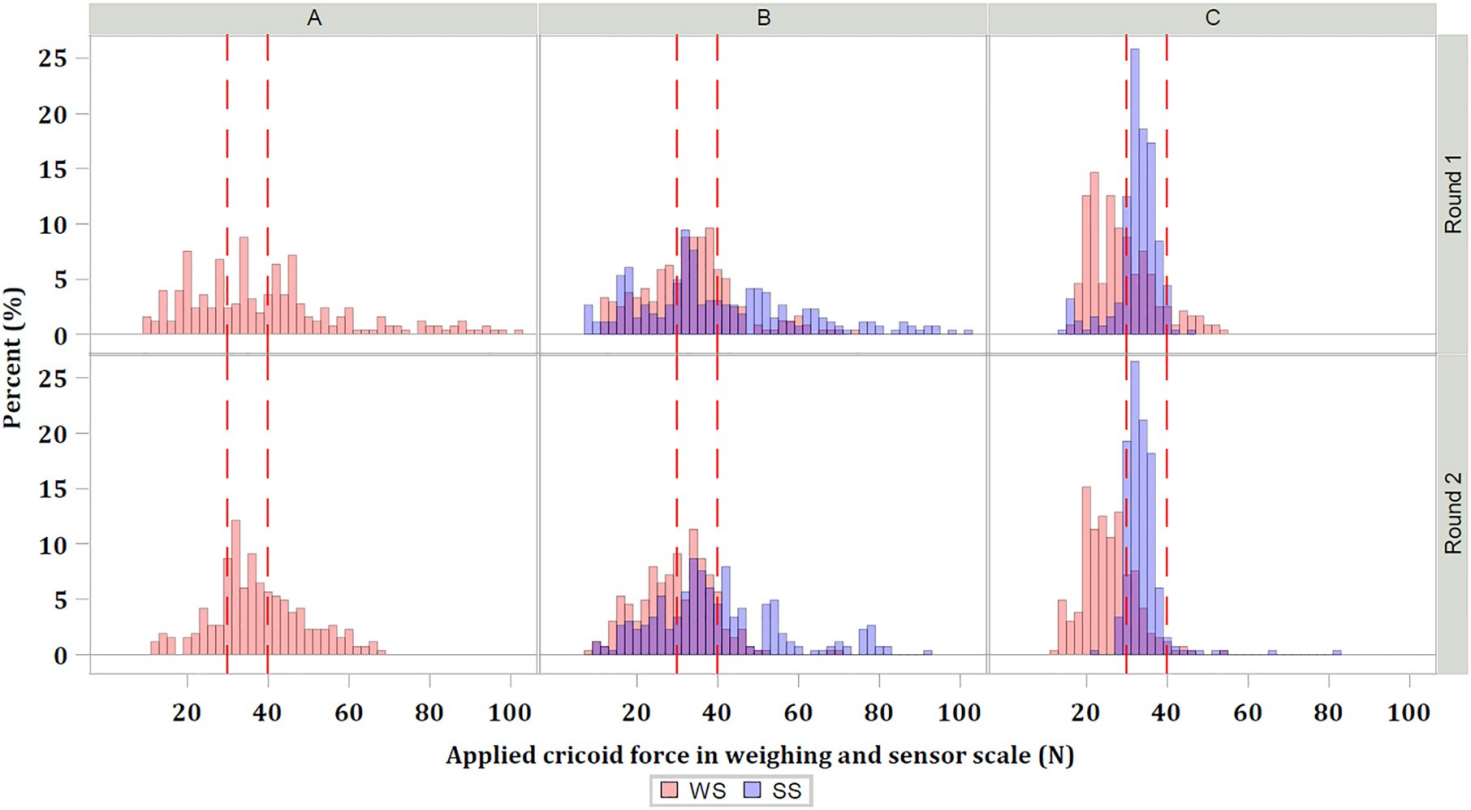
Percentage proportion of force attained within the target range Cricoid pressure measured using weighing scale (WS) and force sensor scale (SS). Red lines denote targeted cricoid pressure range, A denotes Group A, B denotes Group B, C denotes Group C.

Distribution of applied cricoid pressure is illustrated in Fig 3. The narrower distribution of readings and smaller deviation from the targeted range observed in Group C demonstrate increase in consistency of force achieved by anaestheisa nurses in group C.

Significantly higher proportion of target cricoid pressure is achieved with the use of sensor feedback in Group C at 85.9% (95%CI: 82.7%-88.7%) compared to 31.3% (95%CI: 27.4–35.4%) achieved in Group B, where sensor was blinded to the anesthesia nurses.

### Difference between WS and SS

SS reading is higher compared to WS readings for both rounds in both Group B and Group C, see Table 1. The mean difference between WS and SS in Group B is 8.4 Newtons (95% CI: 7.1–9.7 p<0.0001], the mean difference between WS and SS in Group C is 5.8 N (95% CI: 4.5–7.1, p< 0.0001).

The larger difference in readings between WS and SS within groups is demonstrated in Fig 3. The spread of variation between WS and SS is wider in Group B compared to Group C. In Group C, corresponding SS and WS measurements approximate with decreased difference.

## Discussion

This study showed that cricoid pressure when applied blind varied in magnitude and deviated from the target force. Using force sensor and visual feedback, significantly proportion of cricoid pressure (86%) was achieved within the target range. Cricoid force when applied blind exceeded cricoid force applied with the use of biofeedback sensor by a mean of 6–8 Newtons. We feel that this difference is of clinical importance, most clinical studies evaluating cricoid pressure had used a difference of 5 Newtons as an acceptable window [20].

Cricoid pressure is a common practice and a pivotal component during rapid sequence induction of anaesthesia. Reported cricoid pressure applied were found to be inappropriate and variable in the technique used and the amount of force exerted [6–8,10,12,21)]. Even after training, reported retention of cricoid pressure skill ranged from 1 week to 3 months [7, 12,22,23]. Knowledge [9,10,23] of recommended cricoid force was also found to be inappropriate and inadequate in previous studies. Notwithstanding, even with the knowledge of the cricoid force range, it may not be easy to conceptualize force in Newtons and then translate it into practice. Relying on experience in applying the correct force is not reliable as studies had showed no correlation between performance with work experience or profession, rather, strength and dexterity may play a more important role [20,21,24]. Lastly, the consequence of inappropriate application of cricoid pressure is detrimental. Inadequate force results in ineffective cricoid force while excessive force can lead to trauma to oesophagus and airway. Having a real time monitor and biofeedback turns a blind procedure to a guided one, thereby enabling achievement of the target cricoid pressure with reduced variance.

Other findings from this study deserve further mention. Firstly, while sensor visual feedback improved performance, it did not result in 100% target achievement. We postulated that the amount of real-time correction of force (under and over target) and control of force in response to the visual cue maybe influenced by users’ habitual pattern of digital pressure. This was not different from findings in Clayton’s study [6] where desirable cricoid pressure range was achieved among 95% of study participants (anaesthetic nurses and operating department practitioners, recovery staff, intensive care nurses). In his study, Clayton used a floor scale as a guide for force application after simulated training of cricoid pressure on a cricoid model. While the result is encouraging, the multi-task of operator applying cricoid pressure while looking down at the floor scale is impractical and may compromise safety in clinical practice when focus is diverted from patient and the anaesthetist.

Secondly, it is interesting to note that the target force was significantly better achieved in group B (Blinded sensor, fingers on sensor) compared to group A (Blinded, fingers on manikin), both were applied blind. This performance was repeated and demonstrated in both rounds. We believe this is unlikely a result of random variation of practice. Neither is this observation a result of feedback from the investigators as there was no interaction between participants and investigators during the activities where silence was observed strictly. We postulated that direct digital pressure on the rigid interface in force sensor in the group B may have added stability and gave users a better control on the force applied via a tactile feedback.

Thirdly, weighing scale is commonly used in training of cricoid pressure amongst users as well as to verify and quantify the cricoid force in clinical studies that evaluate the efficacy and safety of cricoid pressure [21,25–29]. The assumption that the force transmitted downwards was equivalent to that applied at digital contact has never been challenged. We found a mean difference of 6–10 Newtons between the sensor and weighing scale readings. The reduction in downward weighing force measured by weighing scale may be a result of dissipation of energy arising from compression of structures as well as the vector force coming into play. However, we did not evaluate the angle of force applied by nurses in this study.

One of the earliest reported devices to provide consistent and reproducible cricoid pressure was the cricoid yoke [11] which was described in 1986. The cricoid yoke is made up of 3 essential parts, a pair of elastic "wings" where force is exerted, a moulded cushion for contact with cricoid cartilage and a circuit. The circuit was calibrated to be activated by a contact breaker to give a visual light feedback when a designated force is applied. Good intubating conditions was achieved in 75% (43 out of 57) of parturient undergoing general anaesthesia for elective Caesarean section. In 2015, Taylor [14] described a cricoid pressure compression device to deliver accurate and reproducible cricoid pressure. It takes the form of a moulded plastic yoke with a foam cushion that is applied over the neck and works by providing 2 graded mechanical tactile feedback to the user (at 30 Newtons and 35 Newtons). It was found to decrease the variability of force applied by a mean of 5 Newtons and decreased the upward bias in applied force when evaluated on a cricoid pressure training stimulator. Compared to Taylor’s tactile compression device, Lawe’s cricoid yoke is bulkier, more complex in design and required more training of skill for proper use.

Our study has several limitations. Firstly, the nurses’ performance may be affected by fatigue as they performed the sequential activities from Group A to B to C for 60 seconds duration in each activity. To reduce fatigue, nurses were given a 5 minutes rest between each activity. The results of the performance over time showed that performance was consistent within each group activities and also over time in each activity, suggesting that fatigue is not an important factor in this study. Secondly, the force sensor system is at experimental stage. Bench testing of its performance was carried out on manikin surface, rigid non-human surface, and on wrist, arm, back of hand of human surface, and showed accuracy of 1.5–4 Newtons for our intended force range of 10–40 Newtons. However, it is not sure if the same level of accuracy can be extended to less rigid human structures, such as the neck region of the airway manikin in the study or human neck surface. Thirdly, the same sensor was reused a total of 66 times in the study. The observed repeatability of results of round 1 and round 2 illustrated that the performance of the force sensor system was not affected by the multi-use. Fourthly, our participants were all female. This was because anaesthesia nurses in our medical center are predominantly females. This may however limit the generalizability of findings from our study to male anaesthesia nurses. Lastly, while the force sensor system was designed to be as compact as possible to minimize the interference with working space in the anterior neck region, the system has not taken into account all the ergonomic and human factor in clinical practice, furthermore the study is a simulated scenario with manikin, our results may not be generalized to actual clinical environment. Lastly, while the force sensor maybe of value in the clinical application and maintenance of target cricoid pressure with less variability, the actual ideal cricoid force required to occlude oesophagus is still unknown.

Controversy regarding the efficacy of cricoid pressure in preventing pulmonary aspiration combined with the safety concern of difficult airway associated with poorly applied cricoid pressure (such as difficult mask ventilation, difficult direct laryngoscopy or difficult supraglottic airway device insertion) [30–33] has resulted in ongoing debate about the usefulness of cricoid pressure. Sorbello [34, 35] aptly described the destiny of cricoid pressure seems for now uncertain, however, the fact remains that cricoid pressure is still being widely practice globally. We recognize that discussing cricoid force alone does not address all the controversies that surrounds its use and effectiveness, it is however the first step towards enhancing the performance of cricoid pressure. While a recent review called for training of personnel using technology enhanced cricoid pressure simulation [22]. We proposed the use of technology to enhance cricoid pressure by providing real time objective feedback. Our sensor device may look rudimentary at this stage, with miniaturization it may be replicated for training in manikin or for clinical use.

## Conclusion

Understanding the force applied is the first step to make cricoid pressure a safe procedure. Force sensor visual feedback system enabled application and maintenance of target cricoid pressure with less variability and has potential value in clinical use. The use of weighing scale as a means of quantifying and training cricoid pressure requires further review.
